# Dark Signals in the Choroidal Vasculature on Optical Coherence Tomography Angiography: An Artefact or Not?

**DOI:** 10.1155/2017/5498125

**Published:** 2017-04-28

**Authors:** Rui Hua, Hailin Wang

**Affiliations:** ^1^Department of Ophthalmology, First Hospital of China Medical University, Shenyang, China; ^2^Ophthalmic Clinical Research and Follow-up Center, The Fourth People's Hospital of Shenyang, Shenyang, China; ^3^Department of Ophthalmology, The Fourth People's Hospital of Shenyang, Shenyang, China

## Abstract

Optical coherence tomography angiography (OCTA) based on mathematical processing of sequentially acquired structural OCT images has been applied widely in both retinal and choroidal research and may have advantages over traditional angiography. Images obtained by OCTA are rendered under the assumption that the only moving entity in the retina is blood flow. Optical phenomena and image processing algorithms may create imaging artefacts. Therefore, OCTA images require careful interpretation. This review discusses the dark signals seen in the choroidal vasculature on OCTA using multiple factor analysis. For accurate and comprehensive interpretation of the choroidal vasculature, we recommend simultaneous consideration of the laser light penetration depth and masking effect of retinal pigment epithelium, the orientation of vessels in relation to the scanning lasers and blood flow, the range of regional detectable velocity of blood flow, atrophic tissues in the periphery, and absorption of superior vessels on the scanning laser.

## 1. Introduction

Optical coherence tomography (OCT), including spectral-domain OCT (SD-OCT) and swept-source OCT (SS-OCT), can yield depth-resolved evaluation of reflectance data, together with a volume of three-dimensional information. Using a Fourier transform, the reflections from various depths are frequency-encoded simultaneously [1]. SS-OCT offers potential advantages for documenting the choroid, including not only higher detection efficiency, due to the higher imaging speed, but also an improved imaging range and reduced sensitivity roll-off with imaging depth [2]. OCT angiography (OCTA) using either SD-OCT or SS-OCT assumes that the only moving entity in the retina is blood flow and visualises the vasculature based on motion contrast; moving tissue continuously produces OCTA signals, while stationary tissue produces nearly constant reflection or scattering [3]. For example, using some devices, the motion of erythrocytes is visualised by comparison of sequential cross-sectional scans and generation of a decorrelation map of variability in the speckle pattern [4]. Finally, when the same areas are compared over time on repeat OCT scans, pixels showing changes or fluctuations are regarded as bright, whereas those showing little or no change are regarded as dark. It has been reported that SS-OCTA is better able to demarcate the full extent of choroidal neovascularisation (CNV) than SD-OCTA [5]. Currently, there are many algorithms and methods available for detection of motion contrast, involving amplitude, phase, or a combination of the two in OCT signals.

Nevertheless, OCTA has some advantages and drawbacks when compared with standard angiography. For example, OCTA is unable to visualise accumulation of dye, such as leakage, pooling, and staining, in tissue during standard angiography. Moreover, specific artefacts related to scan acquisition and the physics of optical reconstruction of vascular tissue occur in OCTA [4]. However, traditional angiography has a major limitation in that it cannot image the entire retinal capillary system or visualise new vessels directly and relies on indirect clues such as fluid accumulation or leakage [6]. In contrast, OCTA shows the vascular physiology of the retina [3]. OCTA directly visualises blood vessels in vivo, detects movement of erythrocytes without the need for exogenous dyes, and uses amplitude or phase decorrelation technology with high-frequency and dense volumetric scanning [7].

OCTA has been reported to produce artefacts and requires careful interpretation, because of the inherent complexity of SD-OCT and SS-OCT [3]. Limiting factors include patient cooperation, the time-consuming nature of processing high-resolution images, projection artefacts due to superficial blood flow leading to difficulty in interpreting the networks of blood vessels within the deep retinal layers, and the bulk motion caused by circulating blood and any movement of tissue with respect to the OCT device [8]. Fortunately, axial bulk motion noise is rejected by split-spectrum amplitude-decorrelation angiography [9], which trades some of the axial resolution for decreased noise and higher transverse resolution [10].

It is important to note that there is currently no standard way of evaluating OCTA, and new clinical endpoints need to be established by large studies. In particular, the understanding and interpretation of various signals in the choroidal vasculature on OCTA remains controversial. The imaging mechanism and artefacts mentioned above contribute to the demonstration of blood vessels to some extent. This review investigates and summarises the factors contributing to the dark signals seen in the choroidal vasculature that should be considered when interpreting the results of OCTA

## 2. Penetration Depth of Scanning Lasers and the Masking Effect of Retinal Pigment Epithelium

**Figure 1:**
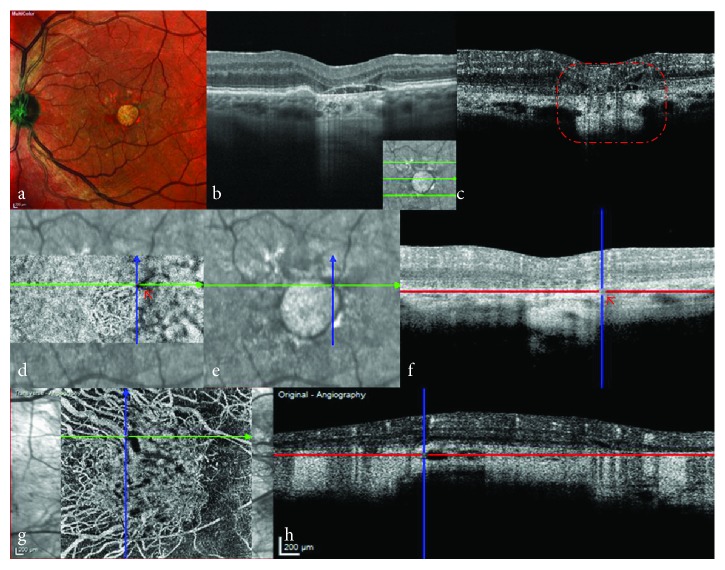
Increased penetration depth of scanning laser in a case of retinal pigment epithelium atrophy. (a) Multicolour images showed a round subfoveal atrophic RPE lesion. (b) SD-OCT shows collapse of the outer retina, atrophy of the choroidal capillaries and RPE, and increased penetration (the inserted image indicates the orientation of OCT scanning). (c) Cross-sectional OCTA at the same location shown in (b) confirms atrophy of the choroidal capillaries and enhances signals in the deep choroidal vasculature (red dotted box). (d) En face OCTA shows a choroidal vessel that is dark because of the masking effect of the RPE and choroidal capillaries (red arrow). (e) An infrared image confirms that the transition between light and dark vascular signal is at the atrophy margin. (f) Cross-sectional OCTA shows a corresponding hypointense halo (red arrow). (g) En face OCTA of another case showing a hypointense halo in the atrophic region with hyperreflective vascular signals. (h) Corresponding cross-sectional OCTA confirming the hyporeflective halo without masked effect, which needs to be explained by other mechanisms. OCTA: Optical coherence tomography angiography; RPE: retinal pigment epithelium; SD-OCT: Spectral-domain optical coherence tomography.

Imaging of the choroid using SD-OCT or SS-OCT is challenging, because the light is scattered and attenuated by the dense choriocapillaris microvasculature and retinal pigment epithelium (RPE) [11]. OCTA, as a depth-resolved examination, requires careful axial segmentation to ensure preservation of important data related to perfused structures and to avoid generation of superimposed images, which are typical of dye angiography [12]. In some cases of RPE atrophy, the enhanced penetration of the scanning laser leads to increased signals from the large choroidal vessels, because of the masking effect of the RPE and removal of the dense microvasculature of the choriocapillaris (Figure 1). However, SS-OCTA, which operates at longer wavelengths, allows better documentation of the choroid, because a better signal is obtained.

## 3. Orientation of Vessels Compared with the Scanning Lasers and Intensity of Blood Flow

**Figure 2:**
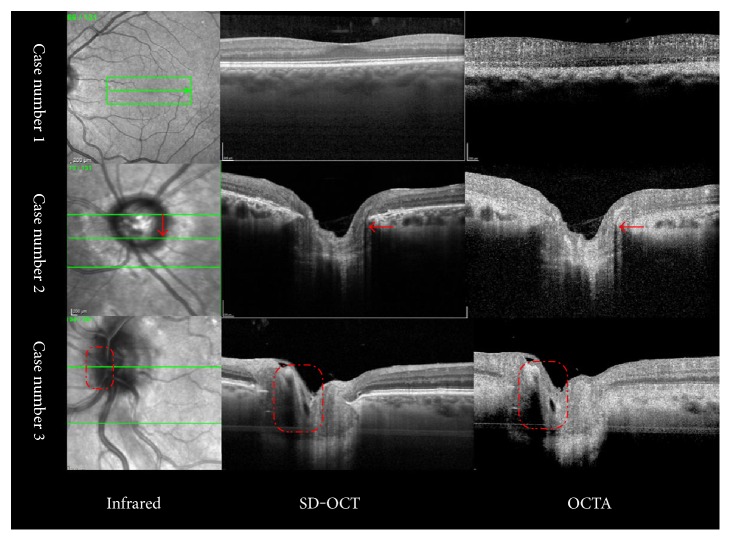
Orientation of vessels according to the type of scanning laser used. The inner and outer neuron layers and the inner and outer photoreceptor segments appear dark on both SD-OCT and OCTA because of the “coherence” of the scanning laser. Similarly, if blood flow is parallel to the orientation of the scanning laser, for example, in the optic disc region, it becomes dark on OCT and OCTA (red arrows and red dotted boxes). OCTA: Optical coherence tomography angiography; SD-OCT: Spectral-domain optical coherence tomography.

OCTA, based on structural OCT data, involves OCT signal amplitude, phase, or a combination of both [3]. One of the characteristics of OCTA is the “coherence” of the scanning laser used. The directional relationship between tissue or blood flow and the scanning laser contributes to the final strength of the signal. This is a common phenomenon in SD-OCT. For example, the inner and outer neuron layers and the inner and outer photoreceptor segments all appear dark on SD-OCT. Usually, blood cells tend to move perpendicular to the OCT light beam and compose OCTA images [13]. However, if blood flow is parallel to the orientation of the scanning laser in the regions of the optic disc and the macula, the image becomes dark on both OCT and OCTA (Figure 2). In OCTA, decorrelation signals above a given threshold, regardless of flow velocity, render bright pixels that are considered to represent blood flow. In contrast, with lower decorrelation signals, static tissues appear as dark pixels. A further increase in flow does not result in increased pixel brightness, indicating saturation. Furthermore, slow blood flow, below a given threshold, will be displayed as dark pixels, and thus vessels will seem to be absent even when they are present [11]. Therefore, choroidal blood flow may appear to be dark, because its intensity does not reach the threshold of the current OCTA devices.

## 4. Range of Detectable Velocity of Regional Blood Flow

**Figure 3:**
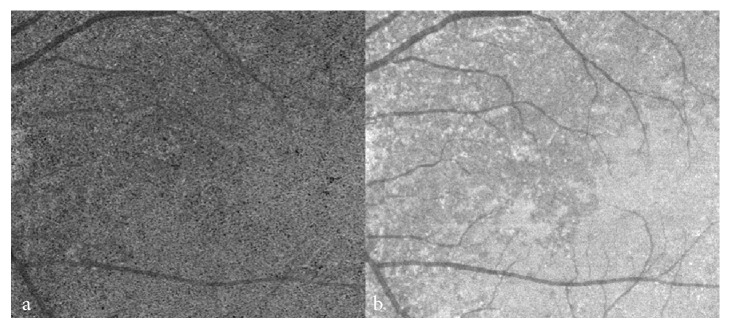
Increased and decreased signals in the choroidal capillaries seen on OCTA. Corresponding en face images for (a) OCTA and (b) SS-OCT. The dark area on the left in (a) and (b) indicates that the velocity of blood flow in the choriocapillaris is slow in the region of choroidal atrophy, compared with that in the bright area. OCTA: Optical coherence tomography angiography; SS-OCT: Swept-source optical coherence tomography.

OCTA only assesses vascular flow within a limited range of dynamic velocity. The ability of OCTA to visualise blood flow is limited to a certain range of flow velocities (minimum 0.5–2 mm/s; saturation 9 mm/s estimated for current devices) [14]. The slowest detectable flow is determined by the time interval between two sequential OCT B-scans, and lesions resulting in flow below the lowest limit of detectable flow cannot be visualised [10]. The decorrelation signal can be more sensitive to slower flows as a result of the increased time interval between consecutive cross-sectional scans [4]. Therefore, this principle should be taken into account in interpretation of OCTA. Dark signals in the choroidal vasculature may be attributable to variable blood flow in this area. It has been reported that the rate of blood flow, determined by the velocity of red blood cell flow, was four times slower in the choriocapillaris than in the inner retinal capillaries in rats [15], which may be attributable to the lack of linear vascular segments in the choriocapillaris, the unusual arrangement of the choroidal microvasculature at right angles to the capillaries, and the oval shape of the capillary lumen [16]. Vascular flow in the choriocapillaris and choroidal microvasculature may occasionally be too slow to generate a decorrelation signal and would preclude documentation of these small vessels on OCTA in some circumstances (Figure 3).

**Figure 4:**
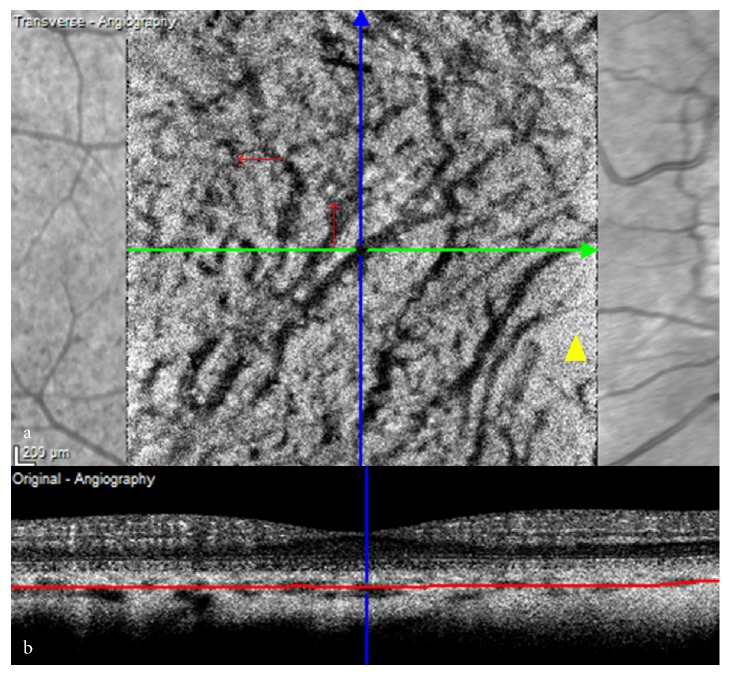
Vascular signals in the middle layer of the choroid. (a) En face OCTA shows cord-like dark vessels attributable to higher blood flow and a hyperreflective choroidal stroma (red arrows) as a result of fringe washout artefact and stromal decorrelation signal. (b) Cross-sectional OCTA confirms a hypointense halo and shows the scanning level. OCTA: Optical coherence tomography angiography.

The relationship between blood velocity in small and large vessels in the retinal tissue and those in the choroid tissue is just the opposite. For example, Bhutto et al. found that choroidal blood flow was higher than retinal blood flow [17]. Similarly, the large vessels may appear to be dark and the stroma may appear to be light (Figure 4), which may be attributed to both fringe washout artefacts related to high blood flow and the limited speed of the imaging system [13]. Additionally, the light choroidal stroma may be due to a stromal decorrelation signal, originating from motion within the stromal tissue, other than the projection artefact of choriocapillaris [13]. Therefore, further investigations are needed to identify the factors affecting the velocity of blood flow.

OCTA also cannot assess vascular permeability without fluorescein or indocyanine green, because leakage during angiography is a diffusion process, rather than a bulk flow phenomenon [3]. The diffusion process is beyond the slowest detectable flow. Interestingly, images obtained by OCTA are not obscured by leakage and clearly show the vessels involved. However, OCTA may also miss the areas of slow blood flow that occur in microaneurysms or fibrotic CNV [10].

**Figure 5:**
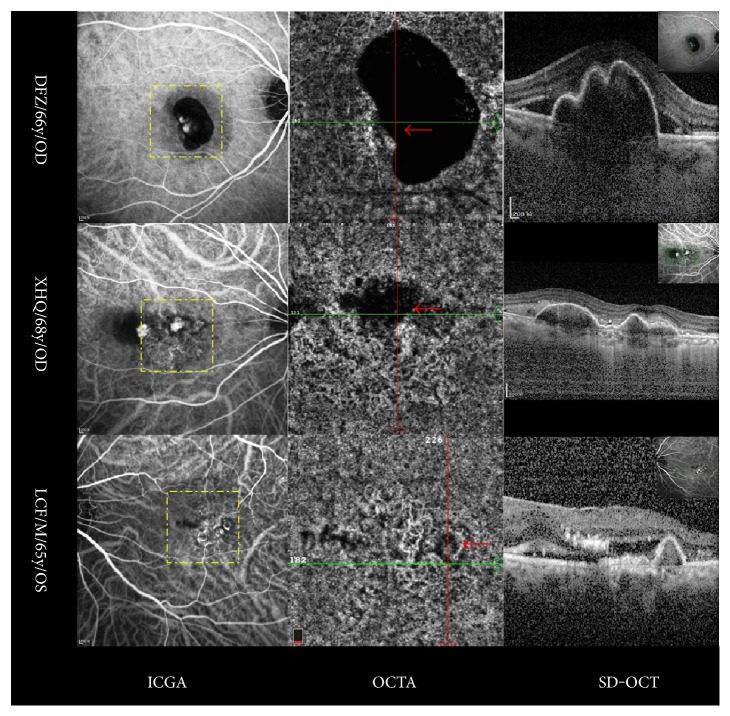
Polypoidal choroidal vasculopathy on OCTA. The round hypoflow appearance of the polyps on OCTA is probably due to unusual blood flow inside the polypoidal lesion (red arrows) in contrast with that in the branching vascular network. This indicates that blood flow is not within the detection limit of the OCTA device (yellow dots on indocyanine green angiography show the range of OCTA; SD-OCT shows both RPE detachment and a double-layer sign; the left side shows anonymised patient information). ICGA: Indocyanine green angiography; OCTA: Optical coherence tomography angiography; RPE: Retinal pigment epithelium; SD-OCT: Spectral-domain optical coherence tomography.

Polypoidal choroidal vasculopathy (PCV) lesions provide another illustration of the range of regional detectable velocity, producing dark abnormal choroidal vessels in OCTA. Segmentation of the choriocapillaris layer in OCTA has revealed the branching vascular network as a hyperflow lesion and a polypoidal lesion as a round hyperflow structure surrounded by a hypointense halo in a few cases, but as a round hypoflow structure in most cases. In contrast with the branching vascular network, the round hypoflow appearance of the polyp probably reflects unusual blood flow inside a polypoidal lesion [18]. Clearly, the absence of signal in a polyp does not mean that there is no blood flow but indicates that blood flow is not within the detection limit of the OCTA device (Figure 5), for example, either increased or decreased flow in the polyps with different orientations and subsequent nonvisualisation of the vascular structure, as discussed above. It has been reported that choroidal blood flow is greater than retinal blood flow [17] and that hemodynamic polypoidal choroidal vasculopathy originates from CNV [19]. Thus, blood flow in polyps is theoretically high, but indocyanine green angiography shows that the polyps do not fill rapidly during early-phase angiography. Therefore, we hypothesise that the apparent absence of an OCTA signal within polypoidal lesions could be due to either the presence of turbulent blood flow inside the polyps or to the fact that blood circulates only at the periphery of the aneurysmal dilation. This hypothesis is strengthened by the fact that the RPE detachment associated with the polypoidal structure also demonstrates an attenuated OCTA signal. Strong signal intensity within the polyp suggests brisk flow and activity [20]. The various structural OCTA aspects of polyps can be explained by the effect of antivascular endothelial growth factor treatment or photodynamic therapy (PDT) on blood flow. Miura et al. [21] have demonstrated that composite Doppler OCT B-scan images show the presence of blood flow inside polypoidal lesions and disappearance or reduction of blood flow after treatment with antivascular endothelial growth factor or PDT, probably due to obstruction of the vessel wall by a thrombus or by a hyalinisation phenomenon [22]. Flow sensitivity is limited by parasitic eye motion, scan intervals, processing techniques, and the threshold set for what is considered to be flow [3]. SS-OCT, with a longer wave-length, provides better documentation of the choroid due to the better signal penetration and reduced washout of interference fringes from flow velocity [14].

## 5. Prominence of Fine Vasculature in the Vicinity of Regional Atrophy

**Figure 6:**
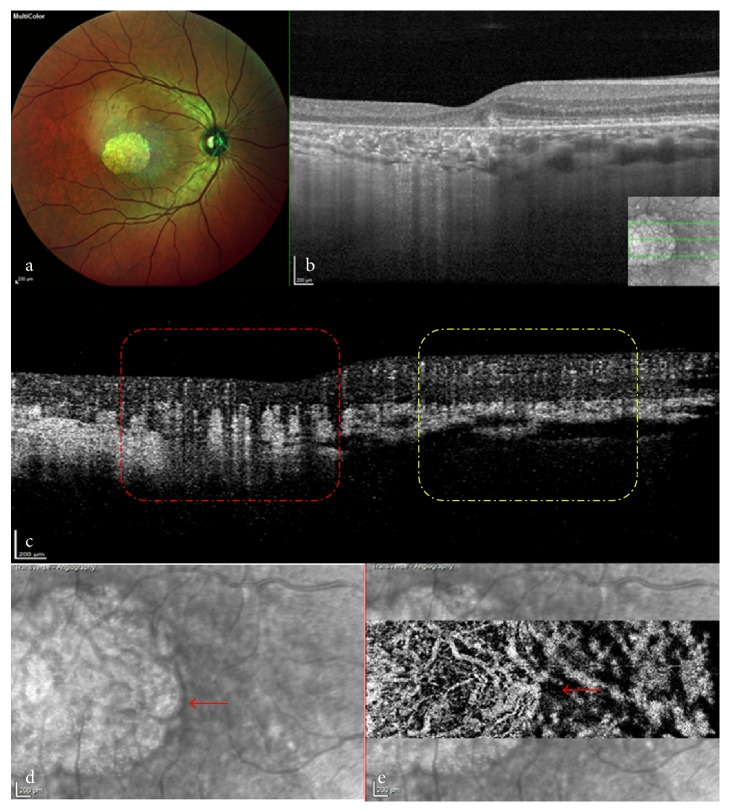
OCTA in a case of outer retinal atrophy. (a) Multicolour image showing a round penetrated hyperreflective region in the macula indicating atrophy of the RPE. (b) SD-OCT showing enhanced choroid in the atrophic RPE area, as compared with the neighbouring normal region (inserted image indicates the direction of OCT scanning). (c) Corresponding cross-sectional OCTA confirms that the remaining fine vessels become obvious with a clear outline after neighbouring regional atrophy, in contrast with the relatively dark background (red dotted box) when compared with normal tissues (yellow dotted box). (d) Infrared image showing the boundary between atrophic and normal areas. (e) En face OCTA shows a hypointense halo beneath the normal RPE and continuing hyperreflective vascular signals due to the masked effect (red arrows in (d) and (e)). OCTA: Optical coherence tomography angiography; RPE: Retinal pigment epithelium; SD-OCT: Spectral-domain optical coherence tomography.

The vascular network of the choriocapillaris includes both anatomical and functional choroidal lobules [23], which are arranged so tightly in the posterior pole that distinct capillary tubes are difficult to identify [4]. Moreover, terminal arterioles or venules from the inner choroid cannot be distinguished from the choriocapillaris because they have the same tomographic appearance [2]. In addition, documentation of the choroid is challenging in both SD-OCT and SS-OCT, because the light is scattered and attenuated by the dense choriocapillaris microvasculature [11]. Furthermore, a morphometric study of normal aging eyes, without degenerative disease, has demonstrated that the density of the choriocapillaris decreases with age [24]. We speculate that after atrophy of the neighbouring vasculature and choroidal stroma, the remaining fine vessels become obvious, with a clear outline, in contrast to the relatively dark background (Figure 6).

## 6. Absorption and Nonreflection of the Scanning Laser

Light passing through a blood vessel can be reflected, refracted, or absorbed. In OCTA, a signal is obtained by reflected light originating from moving blood cells [12]. When incident light passes through moving blood and is therefore not reflected by blood cells, it may be reflected by the underlying tissues [11]. Thus, if the scanning laser penetrates the choroidal blood vessels without successful reflection or if the reflection has been absorbed by superior vessels and tissues, the choroidal blood vessels will appear dark in OCTA images.

## 7. Artefacts

Recently, Bukowska et al. classified OCTA-related artefacts into motion, fringe washout, decorrelation projection, masking and unmasking, and stromal decorrelation signals [13]; dark signals in the choroidal vasculature can also be affected by artefacts. If the movement occurs in the axial direction, because of breathing or arterial pulsation, the entire volumetric OCT will be displaced repeatedly with sufficient decorrelation to cause a change in flow [11]. Each new frame has a lower axial resolution that is less susceptible to axial eye motion caused by blood pulsation [9]. Transverse ocular movements attributable to saccades can cause lateral shearing of the image, introduce white line artefacts on en face images, or create a shift with a gap defect artefact [11], causing the choroidal vessels to appear dark. In contrast to blood cells, which tend to move perpendicular to the OCT light beam, ocular pulsation occurs along the axial direction. The asymmetry in resolution of the OCT system, in which the axial resolution is usually 3 to 5 times higher than the lateral resolution, results in higher sensitivity for tissue motion in the axial than that in transverse direction [13]. If a subject had repeated instances of unstable fixation, the analysis software would mistake these for blood vessels [25] and other vasculatures would be missed. The quilting (checkerboard) defect caused by software correction of ocular movement, in which multiple saccades occurring in different directions, can also cause a rectilinear pattern of distortion, displacement artefacts, and stretch artefacts [11, 26] and may contribute to the darkness of the choroidal vasculature and abnormal signals.

Therefore, focal absence of the choriocapillaris on OCTA may have variable interpretations, including true choriocapillaris atrophy with complete loss of vessels, reduced blood flow, or simply image artefact [4], caused by the technique used for OCT image acquisition, intrinsic characteristics of the eye, eye motion, image processing, and display strategies [10]. Furthermore, displacements caused by involuntary eye movements among repeated B-scans can be compensated for by two-dimensional cross-correlation between two adjacent B-scan images [19].

## 8. Conclusions

Multiple factors are involved in the illustration of choroidal vascular signals in OCTA. In order to obtain an accurate and comprehensive interpretation of the choroidal vasculature in OCTA, we recommend simultaneous consideration of the penetration depth of the scanning laser and masking effect of RPE, the orientation of vessels compared with the scanning laser and the intensity of blood flow, the regional detectable velocity range of blood flow, peripheral atrophic tissues, and absorption of superior vessels on the scanning laser.
